# Sneddon syndrome

**DOI:** 10.11604/pamj.2019.34.9.11903

**Published:** 2019-09-04

**Authors:** Hakima Elmahi, Fatima Zahra Mernissi

**Affiliations:** 1 Department of Dermatology, University Hospital Hassan II, Fez, Morocco

**Keywords:** Sneddon syndrome, skin disease, antiphospholipid antibodies

## Image in medicine

Sneddon's syndrome (SS) is a rare non-inflammatory thrombotic vasculopathy characterized by the combination of cerebrovascular disease with livedo racemose (LR). It has been estimated that the incidence of SS is 4 per 1 million per annum in general population and generally occurs in women between the ages of 20 and 42 years. Approximately 80% of Sneddon's syndrome patients have an antiphospholipid antibody marker. LR may precede the onset of stroke by years and the trunk and/or buttocks are involved in nearly all patients. The cerebrovascular manifestations are mostly secondary to ischemia (transient ischemic attacks and cerebral infarct). Other neurological symptoms range from headache, cerebral hemorrhage, seizures, cognitive and psychiatric disturbances. The involved internal organs include heart, kidney, and eyes. Histological findings of skin are characteristic and the involved vessels are small to medium-sized arteries at the border of dermis to subcutis with a distinct histopathological time course. The main diagnostic criteria are general LR with typical histopathological findings on skin biopsy and focal neurological deficits. The pathogenesis is related to hypercoagulable state and intrinsic small-vessel vasculopathy. The optimal management remains an unsolved problem and long-term anticoagulation have been recommended for cerebral ischemic events based on the presumed pathogenesis. There are controversial results in treatment of SS with immunomodulatory agents. We reported a case of a 49-year old women patient with recurrent ischemic cerebrovascular accidents as the first manifestation, accompanied by livedo reticularis in trunk and limbs and mitral valvulopathy without antiphospholipid antibody.

**Figure 1:**
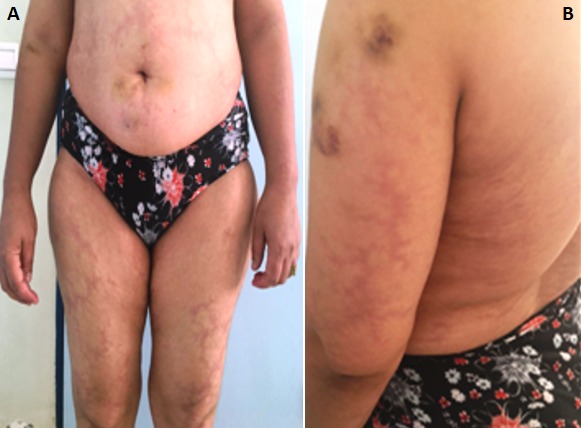
A) livedo reticularis in trunk and limbs; B) livedo reticularis in arms

